# The Ayurveda Education in India: How Well Are the Graduates Exposed to Basic Clinical Skills?

**DOI:** 10.1093/ecam/nep113

**Published:** 2011-02-14

**Authors:** Kishor Patwardhan, Sangeeta Gehlot, Girish Singh, H. C. S. Rathore

**Affiliations:** ^1^Department of Kriya Sharir, Faculty of Ayurveda, Institute of Medical Sciences, Banaras Hindu University, Varanasi 221005, India; ^2^Division of Biostatistics, Department of Community Medicine, Institute of Medical Sciences, Banaras Hindu University, Varanasi, India; ^3^Faculty of Education, Banaras Hindu University, Varanasi, India

## Abstract

“Ayurveda” is an ancient system of healthcare that is native to India. At present, in India, there are more than 240 colleges that offer a graduate-level degree (Bachelor of Ayurvedic Medicine and Surgery—BAMS) in Ayurveda. Even though the Central Council of Indian Medicine, the governing body that monitors the matters related to Ayurveda education, has imposed various educational norms and regulations, the standard of education has been a cause of concern in recent years. The mushrooming of substandard Ayurvedic colleges is the most important factor that is being held responsible for this kind of erosion in the standards. The present study is a mailed survey, which was carried out to evaluate the “Extent of exposure to basic clinical skills during BAMS course” as perceived by the sample groups of students and teachers drawn from 32 Ayurvedic educational institutions spread all over India. A methodically validated questionnaire was used as the tool in the study, to which 1022 participants responded. The study indicates that there are some serious flaws in the existing system of the graduate-level Ayurveda education. Since the Ayurvedic graduates play an important role in the primary healthcare delivery system of the country, governing bodies are required to take necessary steps to ensure the adequate exposure of the students to basic clinical skills. Along with the strict implementation of all the regulatory norms during the process of recognition of the colleges, introducing some changes in the policy model may also be required to tackle the situation.

## 1. Introduction

Ayurveda is the native Indian system of healthcare that is currently used by millions of people in India, Nepal and Sri Lanka for their day-to-day healthcare needs [1]. Though the literature related to Ayurveda is found in the Vedas, it attained a comprehensively documented form known as the “Samhitas” at the turn of the first millennium BC [2]. The “Gurukula” system of education was the method of the Ayurveda training that was generally followed in ancient India. A “Gurukula” was a place where a teacher or “Guru” lived with his family and establishment, and trained the students. *Caraka Samhita*, one of the most popular textbooks on Ayurveda, delineates the process of selecting a suitable textbook and also an appropriate teacher by a disciple besides describing the three ideal methods of learning: self-study, teaching and discussions. The Gurukula system of education suffered a setback during the medieval and colonial periods of Indian history [3]. After the independence, as Ayurveda began to be incorporated into the modern pattern of institutionalization, some of the merits of such an ancient system of education seem to have been compromised.

Today, India officially recognizes Ayurveda and other systems of indigenous medicine along with the conventional biomedicine. To patronize and promote these systems, the Government of India, in 1995, established a separate department for Indian Systems of Medicine and Homeopathy (ISM&H), which is now known as AYUSH (Ayurveda, Yoga, Unani, Siddha, Homeopathy) [4]. Among all the systems in AYUSH, presently Ayurveda holds a prominent position and a major share in the infrastructural facilities in terms of the number of hospitals, dispensaries, educational institutions and registered medical practitioners [5]. The Central Council of Indian Medicine (CCIM), which was established through Indian Medicine Central Council Act of 1970, is the governing body that monitors the matters related to Ayurveda education in India. At present, more than 240 Ayurvedic colleges offer a graduate level degree—“Ayurvedacharya” (Bachelor of Ayurvedic Medicine and Surgery (BAMS)) in India. This course is of 5.5 years' duration after grade 12 with science subjects [6].

Even though the CCIM has imposed various educational norms and regulations, the standard of Ayurvedic education has been a cause of great concern in recent years. The mushrooming of Ayurvedic colleges as a result of the liberal policies of the State Governments and the loopholes in the existing acts are the most important factors that are being held responsible for the erosion in the standards of education [7]. Privatization of the education system is yet another trend that accompanied this phenomenon of mushrooming. For example, at present, the total number of private colleges offering graduate level education in Ayurveda is 186; whereas the number of government colleges offering the same level of education is only 54 [8]. However, most of the undergraduate and postgraduate colleges in the government sector also suffer from a variety of infrastructural constraints [9].

**Figure 1:**
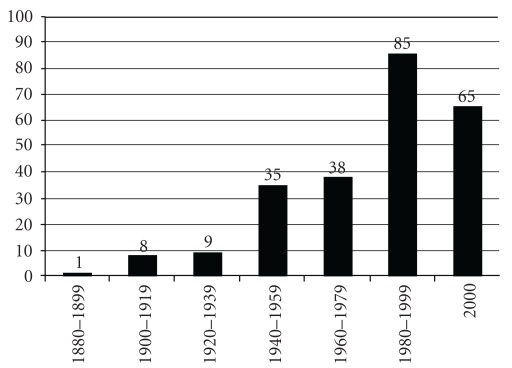
The bar diagram showing the increase in the number of Ayurvedic colleges in India during the last 130 years (based on the data provided by CCIM at http://www.ccimindia.org).

Figure 1 shows the growth in the number of Ayurvedic colleges in India during last 130 years. This diagram is based on the number of the existing colleges and their years of establishment. It is evident from the figure that the majority of these colleges were established during the last 30 years. In many of these colleges, good infrastructure in terms of equipped lecture theatres, laboratories, libraries, operation theatres, *Panchakarma* facilities and adequate number of qualified teaching and non-teaching staff is not available. This has lead to a dilution in the quality of clinical training [9, 10].

## 2. Objectives of the Study

Though some scattered remarks regarding the poor quality of Ayurvedic education are available in some documents and articles, no systematic study has been conducted so far to prove or disprove these assumptions. Keeping this fact in mind, the present study was planned primarily to evaluate the “extent of exposure to basic clinical skills during BAMS course” as perceived by the sample groups of students and teachers drawn from various Ayurvedic educational institutions spread all over India. The secondary objective of this study was to compare the perceptions of the students with those of the teachers to identify the possible differences.

## 3. Study Design

### 3.1. Method Adopted for the Study

Mailed survey was used for this study.

### 3.2. Population

The population for the present study was defined in terms of the students and teachers from Ayurvedic colleges of India during the period of September 2005 to October 2008.

### 3.3. Specific Criteria Considered for Inclusion

The specific criteria considered for inclusion in the study were following:

- All interns/house surgeons registered under BAMS course, who have successfully passed their third professional BAMS examination (held at the end of 4.5 years) in the Ayurvedic colleges recognized by CCIM.
- All post-graduate students registered under “Ayurveda Vachaspati”—MD(Ay) or “Ayurveda Dhanvantari”—MS(Ay) courses in the Ayurvedic colleges recognized by CCIM.
- All teachers of Ayurvedic colleges/universities recognized by CCIM, who possess at least a BAMS or an equivalent degree.

### 3.4. Sample Frame

The sample frame, which became available to us, constituted a list of 242 Ayurvedic colleges spread all over 28 states and 7 union territories of India. As the students and teachers in these colleges formed the primary units of sampling, it was essential to get a list of all these students and teachers for the purpose of randomization. But, as no such database is available in India, we were compelled to accept the list of 242 Ayurvedic colleges as the sample frame for this study.

### 3.5. Sample

With the availability of the sample frame described above, the random cluster sampling technique was considered to be the most appropriate one. Hence, it was decided to include at least 10% of institutions from each geographical zone (North, East, South and West) while trying to include as many states as possible. The colleges from each geographical zone were selected randomly and all the teachers and students from these colleges, as per the operational definition already stated, were taken as the clusters to constitute the sample. Thus, a total of 32 colleges were included in the study.

### 3.6. Preparation of the Preliminary Questionnaire

A preliminary list of items was prepared on the basis of the interactions we had with the students and teachers of several educational institutions. Diverse sources of literature like journals, reports of various committees, national health policy documents, news reports and other articles were referred to for collecting the items. The questionnaire contained multiple statements in simple English language with a uniform negative connotation. The respondents were given the option of recording their responses in the graded form of “Strongly Agree”, “Agree”, “Undecided”, “Disagree” and “Strongly Disagree” by recording a check mark (*✓*) in the respective column provided for the purpose. Apart from these statements, the respondents were also asked to furnish some demographic details such as their age, gender, institutional affiliation and the present status (student or teacher). Students were asked to record the course in which they were enrolled and the teachers were asked to record their academic qualifications, subject specialty and their present designation.

### 3.7. Validation of the Questionnaire for Its Reliability and Consistency

The preliminary questionnaire was distributed to 150 respondents who fulfilled the inclusion criteria in a single institution randomly. The validation process of the questionnaire was carried out on the basis of the first 100 completed questionnaires that we received. The data entry was done using the software “Statistical Package for Social Sciences”, version 11.5 (SPSS Inc., Chicago, IL, USA). Demographic data were fed in the “String” format and the responses to the questionnaire were entered in the “Numerical” format. For the purpose of conversion of responses into the numerical format, the following scoring system was used: Strongly Agree = 5; Agree = 4; Undecided = 3; Disagree = 2; and Strongly Disagree = 1.

The questionnaire was tested for its reliability using Cronbach's coefficient *α*. This was done to find out the correlation between the respective item and the total sum score (without the respective item) and also to find out the internal consistency of the scale (coefficient *α*) if the respective item would be deleted. While validating the scale, value of *α* > 0.7 and item-total correlation >0.2 were considered to be acceptable [11, 12]. Depending on the feedback received, some minor corrections were made in the pattern/language of the statements. Wherever the value of “*α* (when item deleted)” was greater than Cronbach's coefficient *α*, the corresponding item was deleted and the whole process of validation was repeated and thereafter, the questionnaire was finalized. The final questionnaire contained 11 items.  Table 1 shows the items covered in the final questionnaire. Furthermore, the questionnaire contained a copy of confidentiality agreement stating the purpose of the study and assuring strict confidentiality of the respondents. The respondents were asked to sign on a declaration stating that their participation in the study was purely voluntary and the responses given by them were based on their own individual perceptions and that they were not compelled to respond in any particular way by the investigators or by any other authority.

### 3.8. Collection of Data

After validation and testing the questionnaire for its reliability, it was printed on an A4 size paper and was mailed to 32 Ayurvedic colleges. Varying numbers of questionnaires were mailed to different institutions considering the total admission capacity, the total number of teachers present, presence or absence of post-graduate courses, and so forth. The heads of the institutes were requested through a formal letter to distribute the questionnaire among interns, postgraduate students and teachers. A self-addressed stamped envelope was also sent in order to facilitate the return of the completed questionnaires. A period varying from 1 to 2 days was given to the respondents to return the completed questionnaires. The completed questionnaires were then collected and the data entry was carried out as explained earlier.

### 3.9. Response Rate

The response rate for the student group was 59.6% and for the teacher group it was 54%. A total of 1022 participants from 18 states responded to the questionnaire. This number included 644 students and 378 teachers. The majority of participants (195) were from the state of Uttar Pradesh and the least number (19) of them were from the state of Bihar.  Table 2 shows the zone-wise distribution of participants as per their status.

### 3.10. Tabulation and Statistical Tests

The participants were grouped under two categories: “Students” and “Teachers”. Tables of frequency and percentage were framed on the basis of responses to individual items for each group. Independent samples—*T*-test was applied to compare the mean scores of the two groups.

## 4. Observations and Result

Table 1 shows the mean scores for each item (Q1–Q11) for the student group and the teacher group separately. In accordance with the scoring system that was followed in the study, mean scores >3 indicate the tendency towards agreement and those <3 indicate the tendency towards disagreement with the statements. As Table 1 suggests, the mean scores for all the items are >3 for the student group. This indicates a general tendency of the student group towards agreement with all the statements. For all the items except Q11, the mean scores are >3 for the teacher group too. The mean scores of the teacher group for Q11 are 2.83  ±  1.353 indicating a marginal disagreement. Furthermore, on comparing the perceptions of students with those of teachers, the tendency of the student group towards agreement is found to be significantly stronger than the tendency of the teacher group for all items as indicated by “*P*-values”.

## 5. Discussion

The first three items (Q1, Q2 and Q3) in the questionnaire are related to the ability of the Ayurveda graduates to handle the clinical emergencies of the primary level healthcare including infectious diseases and poisoning through Ayurvedic methods. It is to be observed that, both, the student and the teacher groups, show a strong tendency towards agreement with all these three items (mean scores > 4). This means that BAMS graduates are generally not trained to handle clinical emergencies of primary level healthcare through Ayurvedic methods. However, the tendency of the student group towards agreement is significantly stronger than the teacher group for all these three items as indicated by the *P*-values.

The responses to Q4 of the questionnaire show that the extent of clinical exposure to certain basic procedures like incision and drainage, suturing and catheterization is poor in the Ayurvedic educational institutions. Poor exposure of the Ayurveda graduates to conduct normal delivery is another issue that is indicated by the responses to Q5 of the questionnaire.

The reasons for all these perceptions need to be explored. In general, the total number of patients willing to receive Ayurvedic management in the clinical conditions such as those described above may itself be small since the hospitals and clinics offering conventional biomedicine healthcare are becoming increasingly available. This explanation appears to be logical since the participants in the present study, in the form of their responses to Q6 of the questionnaire show a tendency towards agreement that the patients visiting Ayurvedic institutions belong to only a few identifiable categories. These categories include the complaints of joint pain, ano-rectal diseases and neurological manifestations like stroke, and so forth. Therefore, it can be deduced that the students are not exposed to a large variety of cases in the Ayurvedic educational institutions. Another explanation of these observations could be the possible prevalence of cross-prescription. In clinical conditions requiring emergency management, the prescription of antibiotics and other modern biomedicine drugs may be prevalent, at least in a few institutions. However, based on the findings of the present study, no conclusions in this regard can be drawn.

In the present day scenario, a fundamental knowledge of contemporary biomedicine in the subjects like physiology, pathology, biochemistry, pharmacology, medicine, pediatrics, obstetrics & gynecology, ophthalmology, ENT and surgery is essential for all the medical graduates irrespective of their stream. This knowledge is required to understand the reports of laboratory investigations, to evaluate the prognosis and to communicate the Ayurvedic concepts in terms of modern knowledge to those who do not know Ayurveda. Furthermore, the basic knowledge in these areas minimizes the prescription errors due to ignorance. For example, one who knows the implications of liver diseases will not prescribe any herb or other Ayurvedic preparation that might have hepatotoxicity associated with its usage. But, as indicated by the responses to Q7 of the questionnaire, both the teacher and the student group tend to agree that the basic knowledge in these subjects is not being adequately imparted at the BAMS level.

Similarly, the basic knowledge regarding the modern methods of investigation like ECG, X-ray, USG, and so forth, is essential for a BAMS graduate because this helps one in arriving at a correct clinical diagnosis. Furthermore, the right clinical diagnosis determines the line of management and in turn, the final outcome of the treatment. Ancient diagnostic techniques explained in the classical textbooks of Ayurveda are probably inadequate for this age, and therefore, an adequate exposure to basic diagnostic tools is essential at the graduate level. But, as the responses to Q8 of the questionnaire suggest, the Ayurveda graduates are generally not exposed to the basic knowledge of these modern methods of investigation.

A BAMS graduate is required to be trained in areas like genetic counseling, sexual medicine, care of terminally ill patients, geriatrics, and drug and alcohol abuse during the course of study because the patients often seek advice from the practitioners of complementary and alternative medicine (CAM) over these somewhat complicated issues. But, again as the responses to Q9 of the questionnaire imply, the training in these areas is insufficient. A very strong agreement of the student group with this item, as indicated by the mean scores of 4.31  ±  0.894, is a noteworthy point.

Responses to Q10 of the questionnaire indicate that the students are not satisfied with their training in some of the unique skills related to Ayurveda like “Panchakarma” (five major therapeutic procedures: emesis, purgation, two types of medicated enemas and blood letting), “Kshara Sutra” (medicated thread used in the treatment of piles and fistula-in-ano) and “Jalaukavacharana” (leech therapy) and this feedback is too discouraging. The student group (mean score 3.29  ±  1.364) tends to agree with this item more strongly than the teacher group (mean score 3.03  ±  1.338, *P* = .004). The reason for this lapse needs to be looked into seriously. Along with the infrastructural constraints, lack of confidence and willingness among the teachers to carry out these procedures may be some of the other reasons.

The last item, Q11 of the questionnaire is related to the exposure of the students to the basic skills of physical examination, diagnosis and management of common clinical conditions. The responses of the student group indicate that they are not adequately exposed to these skills (mean score 3.11  ±  1.403) rendering them diffident clinicians. On the contrary, the responses of the teacher group to this item tilt towards marginal disagreement (mean score 2.83  ±  1.353) indicating that they tend to perceive the exposure to be adequate and this difference in perceptions is statistically significant (*P* = .002).

Throughout the study, the differences between the perceptions of the student and the teacher groups are found to be statistically significant. While teachers tend to perceive the exposure to basic clinical skills to be relatively better, students consider the same to be inadequate. This observation might be indicating the deterioration in the quality of education over the years.

## 6. Suggested Solutions

The interesting fact is that all the topics related to acquiring the basic clinical skills are included in the curriculum of BAMS course in one form or the other. Also, a generous amount of literature related to toxicology, obstetrics, gynecology, pediatrics, medicine and surgery is available in the classical textbooks of Ayurveda. In addition to this, a lot of time is usually spent on the discussion of the theoretical aspects of Ayurveda in the classrooms [13, 14]. Therefore, the study suggests that the practical application of the theoretical constructs is not taking place adequately. Unless the theory-oriented and textbook-oriented teaching is not transformed into clinically oriented practical training, the problem is probably not going to be solved.

Strict implementation of the regulatory norms while granting approvals to the institutions for running the course can be one of the methods to improve the situation. Some steps are needed to be taken to attract and retain good teachers and clinicians in the education system. The quality of the teachers needs to be ensured by introducing some sort of compulsory qualification screening test in the respective subjects of specialization before declaring them eligible for lectureship. Assessment and accreditation of the institutions by a national level body may also help in improving the quality of educational institutions.

Currently, India follows the policy model of “parallel approach”, where, traditional systems of medicine and modern system of biomedicine are segregated within the national health system. The adaptation of the policy model of “integrated approach”, where, all streams of medicine are integrated at all the levels of medical education and practice, may be the eventual solution for this problem. This model is being followed in some countries such as China and Vietnam and has witnessed a considerable magnitude of success [15, 16]. In addition to this, the idea of practical integration of CAM with the modern biomedicine is gaining momentum in countries like Germany, Italy, Russia and Sweden [17–20]. “Bilateral Education” model can be the other alternative, where, students from one tradition are cross-taught by the experts from the other tradition, imparting knowledge and values in concurrence [21].

## 7. Limitations of the Study

As the total number of institutions covered in the study was only 32, there are chances that a few institutions imparting quality education might have been left out. Also, there was a large regional variation in the number of respondents who participated in the study and therefore, we were unable to figure out the regional differences that might have been prevalent. A similar study involving a large number of institutions might be helpful to substantiate the results of this study.

## 8. Conclusion

The study indicates that there are some serious flaws in the existing system of the graduate level Ayurvedic education. Only a good exposure to basic clinical skills during the medical education can produce a confident physician. Though many topics related to the essential clinical skills are included in the curriculum, the education system has not been able to produce skillful clinicians. Since the Ayurvedic graduates play an important role in the primary healthcare delivery system of the country, this study seeks the attention of governing bodies to take necessary steps ensuring the exposure of the students to the basic clinical skills. Along with the strict implementation of all the regulatory norms during the process of recognition of the colleges, introducing some changes in the policy model may also be required to tackle the situation.

## Figures and Tables

**Table 1:** Items covered in the final questionnaire along with the mean scores of the two groups for each item.

	Item	Group	Mean ± SD	*P*
Q1	Students are not trained to handle the clinical emergencies of primary healthcare level through Ayurvedic methods.	Students Teachers	4.65 ± 0.702 4.35 ± 0.977	.000
Q2	Students are not exposed to any successful Ayurvedic method of primary healthcare in the management of infectious conditions like malaria and tuberculosis.	Students Teachers	4.29 ± 0.981 4.04 ± 1.122	.000
Q3	Students are not exposed to any successful Ayurvedic method of primary healthcare in the management of poisoning.	Students Teachers	4.62 ± 0.721 4.46 ± 0.808	.001
Q4	Students are not exposed sufficiently to the basic clinical skills and procedures like incision and drainage, suturing and catheterization.	Students Teachers	3.48 ± 1.438 3.21 ± 1.387	.004
Q5	Students are not trained sufficiently to conduct normal delivery.	Students Teachers	3.84 ± 1.306 3.32 ± 1.347	.000
Q6	Students are not exposed to a large variety of cases because patients visiting Ayurvedic institutions belong to only few identifiable categories like those complaining of joint pain, ano-rectal diseases, stroke, and so forth.	Students Teachers	3.75 ± 1.232 3.24 ± 1.342	.000
Q7	Students are not exposed sufficiently to the basic modern knowledge of the subjects like physiology, pathology, biochemistry, pharmacology, medicine, pediatrics, obstetrics & gynecology, eye & ENT and surgery.	Students Teachers	3.66 ± 1.287 3.33 ± 1.304	.000
Q8	Students are not exposed sufficiently to the basic skills of interpreting ECG, X-ray and such other diagnostic tools and their clinical utility.	Students Teachers	3.98 ± 1.212 3.70 ± 1.242	.000
Q9	Students are not trained in the basic skills in the areas like genetic counseling, human sexuality, end of life care, geriatrics and drug and alcohol abuse.	Students Teachers	4.31 ± 0.894 3.98 ± 1.104	.000
Q10	Students are not trained sufficiently in the basic clinical methods related to *Panchakarma*, *Kshara Sutra* and *Jalaukavacharana*.	Students Teachers	3.29 ± 1.364 3.03 ± 1.338	.004
Q11	Students are not exposed sufficiently to the basic methods of physical examination, diagnosis and management of common clinical conditions, making them non-confident clinicians/practitioners.	Students Teachers	3.11 ± 1.403 2.83 ± 1.353	.002

The last column shows the results of independent samples *t*-test.

**Table 2:** Zone-wise distribution of participants as per their status.

Zone			Status			Total
	BAMS students	PG students	Lecturers	Readers	Professors	
East	36	51	27	11	6	131
North	84	178	114	34	35	445
South	101	44	63	6	16	230
West	51	99	43	9	14	216

Total	272	372	247	60	71	1022
